# Diversity of Endophytic Yeasts from Agricultural Fruits Positive for Phytohormone IAA Production

**DOI:** 10.3390/biotech11030038

**Published:** 2022-08-25

**Authors:** Aleksey Kachalkin, Anna Glushakova, Rostislav Streletskii

**Affiliations:** 1 Soil Science Faculty, Lomonosov Moscow State University, 119991 Moscow, Russia; 2 G.K. Skryabin Institute of Biochemistry and Physiology of Microorganisms of RAS, 142290 Pushchino, Russia; 3 I.I. Mechnikov Research Institute of Vaccines and Sera, 105064 Moscow, Russia

**Keywords:** endophytic yeasts, IAA, plant beneficial trait, fruits

## Abstract

This study reports the diversity of cultivable endophytic yeasts from agricultural fruits that respond positively to the plant-promoting property of indole-3-acetic acid (IAA) production. The IAA synthesis by the strains was quantified with an Agilent 1100 series liquid chromatography system. IAA was present in the culture liquid of 72% of all 97 strains examined after three days of cultivation. The most active endophytic yeast strains in this study belonged to the species *Aureobasidium pullulans*, *Candida zeylanoides*, *Hanseniaspora uvarum*, *Metschnikowia pulcherrima*, *Meyerozyma caribbica*, *Rhodotorula mucilaginosa*, and *Yarrowia galli*. The highest IAA production was observed in the endophytic strain of *A. pullulans* (9109.19 ± 146.02 μg/g). No significant differences were found between IAA production in strains from agricultural products of different countries. However, the level of IAA production was strictly strain-specific. Our results suggest that the internal tissues of fruits may be a promising source for the isolation of plant-beneficial yeasts that can be used to promote plant growth.

## 1. Introduction

Endophytic yeasts are one of the most promising areas in the study of microbe–plant associations. Plants and yeasts, developing within their internal tissues, together form a single symbiotic system that serves as an excellent model for studying fundamental questions of ecology and evolution, as well as for addressing a number of modern practical questions in agriculture. One of these current practical questions is the search for plant growth-promoting microorganisms (PGPMs) in the diverse range of endophytic yeasts in agriculturally important plants, for example, those that produce phytohormones such as auxins, cytokinins, etc. [1,2,3,4,5]. The effects of strains producing important phytohormones are often responsible for the microbial stimulation of germination, growth and development of higher plants [6,7].

Although many indole compounds in the auxin series have biological activity, IAA is the most potent, widely used and studied in nature. For example, the ascomycete yeasts *Cyberlindnera saturnus* (ex. *Williopsis saturnus*) isolated from the roots of maize could also produce IAA and stimulate growth processes in the plant [8]. Basidiomycete yeasts, *Rhodotorula mucilaginosa,* isolated from poplar and willow were able to produce IAA, which can also promote the growth of some important crops such as corn, tomato, pepper, squash, sunflower, and grasses under nitrogen stress [9]. Thai researchers have extensively studied the phytohormonal activity of epiphytic and endophytic yeasts associated with sugarcane, rice, and other tropical crops. Screening studies indicate a widespread ability of endophytic yeasts to synthesize IAA. The strain dependence of the intensity of the manifestation of this trait is highlighted [6,10,11]. The strain dependence of this trait for the ascomycete yeast *Aureobasidium pullulans* isolated from the phyllosphere and rhizosphere of *Drosera spatulata* is also supported by the results of the study conducted by Taiwanese researchers [12]. The authors also tested the effect of IAA-active strains on the growth of tobacco seedlings (*Nicotiana benthamiana*). It was found that the most active IAA producers stimulated the growth of lateral roots, root hairs, increase in the amount of chlorophyll, elongation of the stem and increase in the number of leaves. No significant effect was found on root length. 

Studies on endophytic yeasts and their phytohormonal activity have been actively conducted for some time and show extremely interesting results [13,14,15,16]. However, they are usually limited to a small sample of strains or a narrow range of plants. In a study of 24 yeast strains from corn roots, 10 strains were able to produce IAA after one week of cultivation [8]. Of the seven yeast strains from mandarins, only four could be reliably confirmed to produce IAA [17]. In another study with endophytic yeasts from mandarins, the activity was detected in all eight strains [18]. In a study with yeasts from grapevine, 67 of the 69 strains were shown to be able to synthesize IAA both without the addition of tryptophan and with this precursor [19]. This prompted us to conduct a large-scale study on the IAA-producing capabilities of endophytic yeast strains isolated from the inner tissues of fruits derived from different countries and to evaluate the potential contribution of endophytic yeasts to stimulate plant growth.

## 2. Materials and Methods

### 2.1. Study Location and Sampling

A study on the synthesis of indole-3-acetic acid (IAA) was carried out for a sample of 97 endophytic yeast strains isolated from fruits of different production purchased from trade networks in the Moscow region (Argentina, Azerbaijan, Belarus, Brazil, China, Chile, Dominican Republic, Egypt, Georgia, Iran, Israel, Kenya, Moldova, Peru, Russia, Serbia, Spain, Turkey, Uzbekistan and Vietnam).

### 2.2. Microbiological Analyses and Species Identification

To study endophytic yeast communities in 2019–2020, fruits were treated according to the following scheme: 70% ethanol, 30 min; 2% sodium hypochlorite, 30 min; 70% ethanol, 30 s; and washing in sterile distilled water for 10 min [20,21]. After the exocarp was removed with a sterile scalpel, the inner tissue was excised, homogenized, and poured with sterile water to obtain a 1:10 dilution. The suspensions were vortexed on a Multi Reax Vortexer (Heidolph Instruments, Germany) for 15 min at 2000 rpm. Three suspensions were prepared for each fruit. The prepared suspensions were plated in three replicates each on glucose-peptone-yeast extract (GPY) agar (20 g/L glucose, 10 g/L peptone, 5 g/L yeast extract, 20 g/L agar) supplemented with chloramphenicol (500 mg/L) to prevent bacterial growth. A total of 3318 plates were incubated at 22 °C for 5–7 days. The grown yeast colonies were classified into morphological types using a dissecting microscope and the number of colonies of each type was counted. From 5 to 7 colonies of each morphotype were isolated in a pure culture. Purified yeast strains were cryopreserved in 10% (*v*/*v*) glycerol in water solution at −80 °C in the yeast collection of the Soil Biology Department at Lomonosov Moscow State University (WDCM 1173). Identification of yeast species was based on the ITS rDNA nucleotide sequence. DNA isolation and PCR were performed according to the procedure described previously [22]. DNA sequencing was performed using the Big Dye Terminator V3.1 Cycle Sequencing Kit (Applied Biosystems, Waltham, MA, USA) with subsequent analysis of the reaction products on an Applied Biosystems 3130xl Genetic Analyzer at the facilities of Evrogen (Moscow, Russia). For sequencing, the ITS5 primer (5’-GGA AGT AAA AGT CGT AAC AAG G) was used [22]. For species identification, nucleotide sequences were compared with those in public databases, using the BLAST NCBI (www.ncbi.nlm.nih.gov (accessed on 7 July 2022)) and the MycoID (www.mycobank.org (accessed on 7 July 2022)) tools. The resulting sequences have been deposited in NCBI (GenBank OP216812-OP216908). The ITS regions of the strains studied were 99.5–100% similar to the type strains. Endophytic strains of 17 yeast species commonly occurring in fruits were examined: *Aureobasidium pullulans* (8 strains); *Candida parapsilosis* (4 strains); *Candida zeylanoides* (7 strains); *Debaryomyces fabryi* (8 strains), *Debaryomyces hansenii* (8 strains), *Filobasidium magnum* (3 strains), *Filobasidium wieringae* (4 strains), *Hanseniaspora uvarum* (7 strains), *Metschnikowia pulcherrima* (9 strains), *Meyerozyma caribbica* (7 strains), *Meyerozyma guilliermondii* (5 strains), *Rhodotorula babjevae* (6 strains), *Rhodotorula mucilaginosa* (8 strains), *Yarrowia deformans* (4 strains), *Yarrowia divulgata* (3 strains), *Yarrowia galli* (3 strains), *Yarrowia lipolytica* (3 strains). Information on the strains examined is presented in Table 1. A full list of the yeasts isolated in the work has been published previously [23]. 

### 2.3. Synthesis of IAA

For IAA synthesis, yeasts were cultured in a liquid medium for 72 h at 22 °C using a Heidolph shaker. An aliquot of 100 µL of the yeast suspension (OD_595_) was added to 20 mL of liquid medium. The medium for culturing the yeasts contained 6.7 g of nitrogenous base (Fluka) and 10 g of glucose per 1 L of water with the addition of tryptophan (1 g/L). 

Sample preparation for the determination of IAA: 20 mL of the culture liquid was acidified to pH = 3 with hydrochloric acid and placed in a 100 mL separatory funnel, to which 20 mL of ethyl acetate [24] was added and shaken vigorously for 1 min. The aqueous phase was then drained and subjected to this procedure again, and the organic phase was placed in a 100 mL evaporation flask. The re-extracted aqueous phase was drained and a new portion of the organic phase was poured into the same flask. The funnel was then washed with 10 mL of ethyl acetate, which was also poured into the flask. The extract was concentrated on a rotary evaporator (50 rpm) to a final volume of ≤0.5 mL [25]. The resulting concentrate was transferred to a 1.5 mL chromatography vial, and 0.5 mL of acetonitrile was added to the evaporation flask and placed in an ultrasonic bath for 1 min to separate the IAA from the flask walls. The acetonitrile from the flask was also transferred to the vial. Then, 0.5 mL of acetonitrile was again added to the flask and the treatment was repeated. If necessary, the contents of the vial were made up to 1.5 mL of acetonitrile. Quantification of IAA was performed using an Agilent 1100 series high performance liquid chromatograph with UV detector. A Security Guard Catridges C18 4 ×3.0 mm precolumn and a Diasfer 110-C18 5 µm 4.0 × 250 mm analytical HPLC column were used. The detection wavelength was 222 nm. Flow rate of the eluent—1.0 mL/min. Mobile phase—water, acetonitrile, 0.05% trifluoroacetic acid (45:54:1% *v*/*v*). The volume of the injected sample was 10 µL. The temperature of the column thermostat was 25 °C. The analysis was performed for 18 min.

Solutions of the standard substance indole-3-acetic acid (DiaM) in acetonitrile were used to calibrate the instrument. 

IAA produced by yeasts was expressed as per liter and per gram of dry biomass.

**Figure 1:**
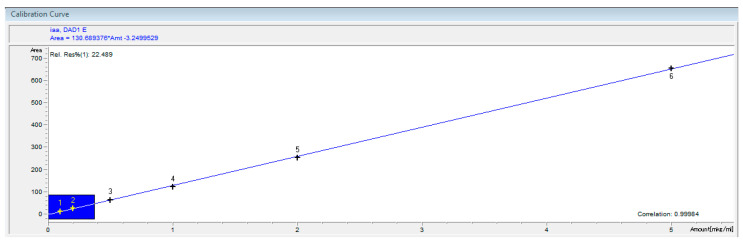
Calibration curve for standard solutions of IAA.

Calibration was performed in six steps (Figure 1). The correlation coefficient r > 0.995. The biomass was used to calculate the specific auxin concentration [26,27]. For each strain, the study was repeated twice.

### 2.4. Data Analyses

Statistical data processing and graphical presentation of the obtained results were carried out using Excel 2010 (Microsoft, Albuquerque, NM, USA) and Statistica 8.0 (StatSoft, Tulsa, OK, USA) programs. The analysis of variance (ANOVA) was carried out for groups comparison. Statistical significance was judged at the level of *p* < 0.05. 

## 3. Results

Indole-3-acetic acid was present in the culture liquid of 69 (72% of all strains examined) of the 97 endophytic yeast strains studied after three days of cultivation (Table 1).

**Figure 2:**
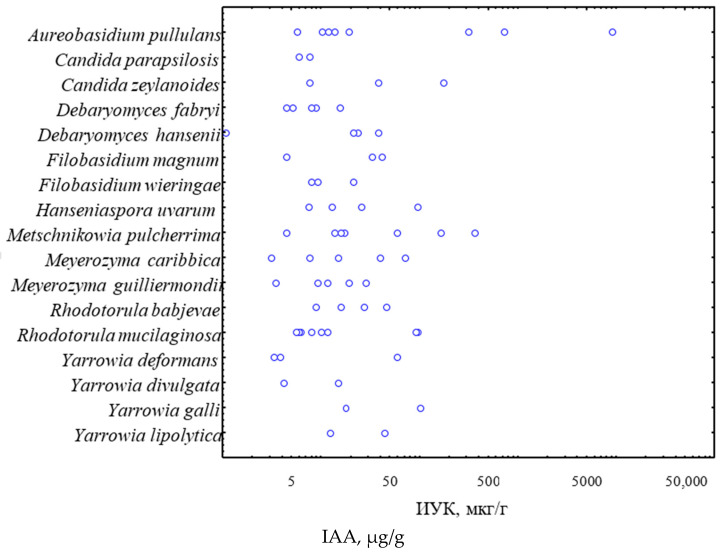
IAA production by the strains of the studied yeast species (logarithmic scale).

This value of active strains differs from the data we previously obtained for non-endophytic yeast strains from different natural substrates. At that time, the percentage of active strains was 92% [27,28]. The observed differences can be explained by the different approaches used in the analysis: in the current study, the determination of indole-3-acetic acid in the culture liquid was performed without the step of pre-concentration and the minimum values of IAA synthesis were not considered. In addition, we were interested in the rapid response of the yeasts and the ability to produce IAA in significant amounts. However, it is known that for some strains the maximum concentration of IAA in the culture liquid is reached only on day 5–7 [17]. The results we obtained for strains of the most abundant yeast species in agricultural products show that all yeast species studied are capable of synthesizing IAAs, but the extent of production is strictly strain-specific (Table 2 and Figure 2).

Maximum IAA production (9109.2 μg/g) was found in strain *A. pullulans* (YE-0979) Strains of this species are regularly cited as the most active producers of IAA in various studies [12,29]. In our previous study on the phytohormonal activity of non-endophytic yeasts, we found the maximum IAA production (7990.4 µg/g) in strain *Metschnikowia pulcherrima* Y-5623 [27]. In this study, the endophytic strains of this ascomycetous yeast species also showed high IAA activity.

A comparison of the yeast groupings studied, such as Phylum and Origin, showed no statistically significant results. It is most likely that the ability of endophytic yeast to synthesize IAA is determined by the nature of the strains.

## 4. Discussion

It is widely recognized that endophytic yeasts have an excellent ability to promote plant growth, which can be a boon to agricultural practices. This ability of endophytic yeasts is based on their ability to secrete bioactive compounds such as auxins, gibberellins, siderophores [30,31]. The production of plant hormones provides a direct method of promoting plant growth by endophytes. Auxins and gibberellins have many growth-promoting properties in plants, including promotion of root growth and stem elongation and, more broadly, cell proliferation and elongation. IAA has also been shown to play a role in controlling fungal diseases [31,32]. In particular, the production of IAA by endophytic yeasts has been reported and discussed in detail by several groups [5,8,30]. Our screening of strains from the internal tissues of fruits from different countries shows that more than 70% of endophytic yeast strains produce a significant amount of IAA relatively quickly, i.e., within the first 72 h after cultivation. No significant differences were found between the production of IAA by strains from agricultural products from different countries (Table 1). However, our previous studies have shown that tropical strains of ascomycete yeasts have significantly higher phytohormonal activity compared to strains from other regions [27,28]. Our results suggest that endophytic yeast complexes from the internal tissues of fruits may be a promising source of plant-beneficial yeast strains that can be used to promote plant growth. The isolation of an opportunistic yeast species, *Candida parapsilosis*, deserves separate consideration. The studied endophytic strains of this species showed the lowest IAA synthesis property. Previously, we detected *C. parapsilosis* yeasts in high abundance in the internal tissues of ripe fruits of apples and pears growing in conditions of high anthropogenic stress [22,23]. Most likely, opportunistic yeasts belong to the species contaminating agricultural products. This is indirectly indicated by their weak ability to synthesize the phytohormone IAA. 

IAA biosynthesis by endophytic yeasts from different fruits is highly strain-specific. Further detailed studies are planned to investigate the multiple factors affecting gene expression of IAA biosynthesis to varying degrees at both species and strain levels.

## Figures and Tables

**Table 1:** IAA production (with standard deviations) of studied strains and information about it.

Strain KBP	Species	Phylum	Country of Origin	Substrate	IAA, mg/L	IAA, μg/g
YE-0002	*Candida zeylanoides*	ASC *	Turkey	nectarine	-	-
YE-0007	*Debaryomyces hansenii*	ASC	Turkey	nectarine	-	-
YE-0013	*Metschnikowia pulcherrima*	ASC	Turkey	cherry	0.1 ± 0.01	15.85 ± 2.16
YE-0017	*Debaryomyces hansenii*	ASC	Iran	pepper	0.01 ± 0.00	1.09 ± 0.24
YE-0024	*Hanseniaspora uvarum*	ASC	Russia	gooseberry	-	-
YE-0031	*Metschnikowia pulcherrima*	ASC	Russia	apple	0.07 ± 0.01	17.15 ± 2.51
YE-0034	*Filobasidium wieringae*	BAS	Turkey	cherry	-	-
YE-0043	*Hanseniaspora uvarum*	ASC	Iran	grapes	0.05 ± 0.01	7.53 ± 1.25
YE-0045	*Meyerozyma caribbica*	ASC	Brazil	mango	0.29 ± 0.24	40.24 ± 33.14
YE-0053	*Filobasidium wieringae*	BAS	Moldova	cherry	0.03 ± 0.00	21.32 ± 1.15
YE-0058	*Yarrowia galli*	ASC	Russia	apple	0.11 ± 0.00	17.85 ± 0.03
YE-0061	*Yarrowia galli*	ASC	Argentina	apple	-	-
YE-0065	*Rhodotorula babjevae*	BAS	Russia	apple	0.08 ± 0.00	16.08 ± 0.00
YE-0066	*Rhodotorula babjevae*	BAS	Argentina	apple	0.05 ± 0.00	8.78 ± 0.89
YE-0067	*Yarrowia lipolytica*	ASC	Russia	apple	-	-
YE-0068	*Yarrowia galli*	ASC	Turkey	apple	0.63 ± 0.02	100.60 ± 4.75
YE-0069	*Filobasidium wieringae*	BAS	Russia	apple	0.06 ± 0.01	7.86 ± 1.30
YE-0070	*Hanseniaspora uvarum*	ASC	Russia	apple	0.11 ± 0.03	25.34 ± 3.90
YE-0071	*Yarrowia divulgata*	ASC	Russia	apple	-	-
YE-0072	*Candida zeylanoides*	ASC	Chile	apple	0.08 ± 0.01	37.57 ± 1.66
YE-0073	*Candida zeylanoides*	ASC	Russia	apple	-	-
YE-0074	*Rhodotorula mucilaginosa*	BAS	Russia	apple	0.54 ± 0.09	91.74 ± 13.51
YE-0079	*Candida zeylanoides*	ASC	Russia	apple	-	-
YE-0080	*Yarrowia lipolytica*	ASC	Russia	apple	0.08 ± 0.01	12.35 ± 1.08
YE-0081	*Yarrowia divulgata*	ASC	Russia	gooseberry	0.08 ± 0.01	14.85 ± 3.00
YE-0086	*Yarrowia lipolytica*	ASC	Russia	apple	0.26 ± 0.05	44.61 ± 7.70
YE-0093	*Yarrowia deformans*	ASC	Russia	apple	0.31 ± 0.06	58.33 ± 11.56
YE-0106	*Hanseniaspora uvarum*	ASC	Russia	apple	0.04 ± 0.00	12.94 ± 0.03
YE-0114	*Yarrowia divulgata*	ASC	Russia	gooseberry	0.03 ± 0.00	4.15 ± 0.14
YE-0118	*Candida parapsilosis*	ASC	Russia	beet	0.03 ± 0.01	7.63 ± 3.10
YE-0119	*Yarrowia deformans*	ASC	Russia	apple	-	-
YE-0120	*Rhodotorula babjevae*	BAS	Russia	beet	-	-
YE-0122	*Candida parapsilosis*	ASC	Russia	apple	0.01 ± 0.00	5.97 ± 0.38
YE-0125	*Yarrowia deformans*	ASC	Belarus	pepper	0.02 ± 0.00	3.31 ± 0.06
YE-0128	*Yarrowia deformans*	ASC	Egypt	tangerine	0.02 ± 0.00	3.85 ± 0.05
YE-0130	*Meyerozyma guilliermondii*	ASC	Egypt	tangerine	0.02 ± 0.00	3.41 ± 0.19
YE-0131	*Candida zeylanoides*	ASC	Russia	apple	0.06 ± 0.00	7.67 ± 0.13
YE-0133	*Filobasidium magnum*	BAS	Russia	apple	0.18 ± 0.02	41.04 ± 4.55
YE-0139	*Metschnikowia pulcherrima*	ASC	Russia	apple	-	-
YE-0151	*Debaryomyces hansenii*	ASC	Spain	tangerine	0.15 ± 0.01	38.44 ± 2.23
YE-0159	*Metschnikowia pulcherrima*	ASC	Russia	quince	0.22 ± 0.03	366.21 ± 24.36
YE-0164	*Rhodotorula mucilaginosa*	BAS	Serbia	plum	0.04 ± 0.00	5.86 ± 0.78
YE-0166	*Rhodotorula mucilaginosa*	BAS	Russia	apple	0.04 ± 0.00	7.9 ± 0.41
YE-0177	*Rhodotorula mucilaginosa*	BAS	Russia	pea	0.03 ± 0.00	6.1 ± 0.05
YE-0179	*Meyerozyma caribbica*	ASC	Dominican Republic	coconut	0.51 ± 0.06	71.42 ± 7.22
YE-0180	*Debaryomyces hansenii*	ASC	Russia	tomato	0.13 ± 0.01	23.76 ± 0.2
YE-0204	*Meyerozyma caribbica*	ASC	Iran	kiwi	0.02 ± 0.00	3.13 ± 0.08
YE-0205	*Rhodotorula mucilaginosa*	BAS	Iran	kiwi	0.05 ± 0.01	11.35 ± 1.79
YE-0214	*Debaryomyces hansenii*	ASC	Russia	pepper	-	-
YE-0216	*Hanseniaspora uvarum*	ASC	Russia	apple	-	-
YE-0217	*Candida zeylanoides*	ASC	Russia	apple	-	-
YE-0220	*Rhodotorula babjevae*	BAS	Russia	carrot	-	-
YE-0221	*Filobasidium wieringae*	BAS	Russia	tomato	0.04 ± 0.01	9.09 ± 0.86
YE-0230	*Filobasidium magnum*	BAS	Israel	persimmon	0.12 ± 0.02	33.42 ± 5.34
YE-0242	*Aureobasidium pullulans*	ASC	Peru	mango	0.75 ± 0.05	736.80 ± 37.48
YE-0250	*Metschnikowia pulcherrima*	ASC	Russia	strawberry	0.05 ± 0.01	60.11 ± 9.85
YE-0256	*Aureobasidium pullulans*	ASC	Serbia	cherry	0.11 ± 0.03	19.35 ± 4.5
YE-0260	*Aureobasidium pullulans*	ASC	Turkey	apricot	0.43 ± 0.02	319.26 ± 7.01
YE-0269	*Aureobasidium pullulans*	ASC	Peru	mango	0.06 ± 0.06	10.33 ± 10.33
YE-0270	*Aureobasidium pullulans*	ASC	Turkey	apricot	0.04 ± 0.00	5.84 ± 0.61
YE-0282	*Filobasidium magnum*	BAS	Turkey	grapes	0.02 ± 0.00	4.48 ± 1.00
YE-0289	*Aureobasidium pullulans*	ASC	Russia	currants	0.08 ± 0.01	13.74 ± 1.30
YE-0299	*Rhodotorula babjevae*	BAS	Russia	apple	0.09 ± 0.00	27.32 ± 0.84
YE-0302	*Hanseniaspora uvarum*	ASC	Russia	apple	-	-
YE-0303	*Metschnikowia pulcherrima*	ASC	Russia	apple	0.57 ± 0,01	163.87 ± 38.82
YE-0310	*Hanseniaspora uvarum*	ASC	Azerbaijan	persimmon	0.06 ± 0.01	96.13 ± 4.20
YE-0316	*Metschnikowia pulcherrima*	ASC	Israel	persimmon	0.07 ± 0.01	13.97 ± 1.54
YE-0337	*Candida zeylanoides*	ASC	Azerbaijan	persimmon	1.32 ± 0.21	176.43 ± 30.64
YE-0347	*Debaryomyces hansenii*	ASC	Georgia	pistachios	-	-
YE-0367	*Candida parapsilosis*	ASC	Vietnam	banana	-	-
YE-0503	*Meyerozyma caribbica*	ASC	Vietnam	jackfruit	0.1 ± 0.01	14.55 ± 1.55
YE-0623	*Candida parapsilosis*	ASC	Vietnam	passion fruit	-	-
YE-0625	*Meyerozyma caribbica*	ASC	Vietnam	passion fruit	0.05 ± 0.01	7.82 ± 1.46
YE-0652	*Metschnikowia pulcherrima*	ASC	Vietnam	tangerine	0.03 ± 0.00	4.49 ± 0.08
YE-0672	*Debaryomyces fabryi*	ASC	Russia	walnut	-	-
YE-0676	*Debaryomyces fabryi*	ASC	Egypt	tangerine	0.03 ± 0.00	5.14 ± 0.37
YE-0678	*Debaryomyces fabryi*	ASC	Chile	apple	-	-
YE-0680	*Debaryomyces fabryi*	ASC	Chile	kiwi	0.07 ± 0.01	15.56 ± 1.96
YE-0681	*Debaryomyces fabryi*	ASC	Turkey	grapes	0.03 ± 0.00	4.45 ± 0.15
YE-0684	*Debaryomyces fabryi*	ASC	Georgia	peanuts	0.04 ± 0.01	8.95 ± 2.24
YE-0688	*Debaryomyces fabryi*	ASC	Turkey	tomato	0.04 ± 0.01	7.83 ± 1.91
YE-0700	*Meyerozyma guilliermondii*	ASC	Egypt	orange	0.07 ± 0.02	9.2 ± 2.02
YE-0712	*Meyerozyma guilliermondii*	ASC	Egypt	orange	0.15 ± 0.02	28.57 ± 5.10
YE-0713	*Debaryomyces fabryi*	ASC	Spain	tangerine	-	-
YE-0718	*Debaryomyces hansenii*	ASC	Spain	tangerine	-	-
YE-0719	*Debaryomyces hansenii*	ASC	Turkey	apple	0.11 ± 0.00	21.71 ± 0.53
YE-0721	*Rhodotorula babjevae*	BAS	Turkey	apple	0.47 ± 0.06	45.29 ± 3.94
YE-0722	*Meyerozyma guilliermondii*	ASC	Vietnam	longan	0.06 ± 0.01	11.56 ± 0.95
YE-0725	*Metschnikowia pulcherrima*	ASC	Vietnam	passion fruit	-	-
YE-0728	*Meyerozyma guilliermondii*	ASC	Vietnam	longan	0.07 ± 0.00	18.87 ± 0.00
YE-0735	*Meyerozyma caribbica*	ASC	Vietnam	guava	-	-
YE-0878	*Meyerozyma caribbica*	ASC	Iran	watermelon	-	-
YE-0882	*Rhodotorula mucilaginosa*	BAS	Iran	melon	0.05 ± 0.00	9.87 ± 0.51
YE-0959	*Rhodotorula mucilaginosa*	BAS	Israel	watermelon	0.03 ± 0.00	5.5 ± 0.18
YE-0967	*Rhodotorula mucilaginosa*	BAS	Israel	watermelon	0.61 ± 0.08	94.98 ± 19.35
YE-0979	*Aureobasidium pullulans*	ASC	Israel	cress	14.96 ± 1.73	9109.19 ± 146.02
YE-1002	*Aureobasidium pullulans*	ASC	Uzbekistan	parsley	0.05 ± 0.05	11.98 ± 11.98

* ASC—Ascomycota; BAS—Basidiomycota.

**Table 2:** Proportion of active strains of the studied endophytic yeast species and average IAA production (with standard deviations) in the culture liquid and per unit biomass.

Yeast Species	Proportion (%) of Strains Starting to Synthesize IAA after 72 h	IAA, mg/L	IAA, μg/g Dry Biomass
*Aureobasidium pullulans*	100	2.06 ± 1.27	1278.31 ± 766.88
*Candida parapsilosis*	50	0.01 ± 0.01	3.40 ± 1.43
*Candida zeylanoides*	42.9	0.22 ± 0.14	34.10 ± 18.25
*Debaryomyces fabryi*	62.5	0.03 ± 0.01	5.24 ± 1.36
*Debaryomyces hansenii*	50	0.05 ± 0.02	10.62 ± 3.67
*Filobasidium magnum*	100	0.11 ± 0.03	26.31 ± 7.28
*Filobasidium wieringae*	75	0.03 ± 0.01	9.56 ± 2.91
*Hanseniaspora uvarum*	57.1	0.04 ± 0.01	20.28 ± 8.93
*Metschnikowia pulcherrima*	77.8	0.12 ± 0.04	71.29 ± 28.21
*Meyerozyma caribbica*	71.4	0.14 ± 0.06	19.59 ± 7.75
*Meyerozyma guilliermondii*	100	0.07 ± 0.02	13.82 ± 3.32
*Rhodotorula babjevae*	66.7	0.14 ± 0.06	19.87 ± 5.88
*Rhodotorula mucilaginosa*	100	0.18 ± 0.06	29.16 ± 9.83
*Yarrowia deformans*	75	0.09 ± 0.05	16.37 ± 9.43
*Yarrowia divulgata*	66.7	0.04 ± 0.02	7.60 ± 3.20
*Yarrowia galli*	66.7	0.25 ± 0.12	39.49 ± 19.64
*Yarrowia lipolytica*	66.7	0.12 ± 0.05	18.99 ± 8.65

## Data Availability

The data presented in this study are available on request from the corresponding author.
